# Automated rotator cuff tear classification using 3D convolutional neural network

**DOI:** 10.1038/s41598-020-72357-0

**Published:** 2020-09-24

**Authors:** Eungjune Shim, Joon Yub Kim, Jong Pil Yoon, Se-Young Ki, Taewoo Lho, Youngjun Kim, Seok Won Chung

**Affiliations:** 1 Center for Bionics, Korea Institute of Science and Technology, Seoul, 02792 Korea; 2 Department of Orthopedic Surgery, Yeson Hospital, Bucheon, 14555 Korea; 3 Department of Orthopaedic Surgery, School of Medicine, Kyungpook National University, Daegu, 41944 Korea; 4 Department of Orthopaedic Surgery, Center for Shoulder and Elbow Surgery, Konkuk University School of Medicine, Seoul, 143-729 Korea

**Keywords:** Translational research, Biomedical engineering

## Abstract

Rotator cuff tear (RCT) is one of the most common shoulder injuries. When diagnosing RCT, skilled orthopedists visually interpret magnetic resonance imaging (MRI) scan data. For automated and accurate diagnosis of RCT, we propose a full 3D convolutional neural network (CNN) based method using deep learning. This 3D CNN automatically diagnoses the presence or absence of an RCT, classifies the tear size, and provides 3D visualization of the tear location. To train the 3D CNN, the Voxception-ResNet (VRN) structure was used. This architecture uses 3D convolution filters, so it is advantageous in extracting information from 3D data compared with 2D-based CNNs or traditional diagnosis methods. MRI data from 2,124 patients were used to train and test the VRN-based 3D CNN. The network is trained to classify RCT into five classes (None, Partial, Small, Medium, Large-to-Massive). A 3D class activation map (CAM) was visualized by volume rendering to show the localization and size information of RCT in 3D. A comparative experiment was performed for the proposed method and clinical experts by using randomly selected 200 test set data, which had been separated from training set. The VRN-based 3D CNN outperformed orthopedists specialized in shoulder and general orthopedists in binary accuracy (92.5% vs. 76.4% and 68.2%), top-1 accuracy (69.0% vs. 45.8% and 30.5%), top-1±1 accuracy (87.5% vs. 79.8% and 71.0%), sensitivity (0.92 vs. 0.89 and 0.93), and specificity (0.86 vs. 0.61 and 0.26). The generated 3D CAM provided effective information regarding the 3D location and size of the tear. Given these results, the proposed method demonstrates the feasibility of artificial intelligence that can assist in clinical RCT diagnosis.

## Introduction

Convolutional neural networks (CNN) have frequently been shown to be successful at image classification. With the considerable growth in hardware performance such as graphics processing units (GPUs) and availability of big data, deep-learning-based image processing methods have outperformed traditional methods since 2012, when Alexnet^1^ demonstrated results far beyond existing methods in the ImageNet^2^ competition. CNN is an essential method for automatic image classification and also exhibits excellent performance in other image processing tasks such as object detection^3^ and segmentation^4^. Because of this, deep-learning-enhanced algorithms can be employed for medical image processing. Researchers have proposed CNN-based detection or segmentation^5^ of organs in medical images. CNN-based classification methods can be useful in computer-aided diagnosis, which can lead to greater accuracy and reliability in medicine. In particular, CNN architecture such as inception^6,7^ has shown specialist-level performance in classifying skin cancer^8^, shoulder fracture detection^9^, and diabetic retinopathy^10^ from 2D images of the affected area.

However, much of the existing research on diagnosis using classification methods have limitations. First, the preprocessing of learning data requires the time-consuming manual labor of clinical experts. Most CNN classification-based diagnosis methods are based on separating 2D slice images into patches. Although 3D CNN-based diagnosis methods have been tried, they use relatively a narrow and simple CNN architecture^11^. Instead, they require precise localization or segmentation from raw images, which is also tedious task. Second, most of the previous methods have focused on only 2D-based CNNs. Unlike the RGB images used for classifying skin cancer or diabetic retinopathy, 3D medical images such as those from computed tomography (CT) or magnetic resonance imaging (MRI) are widely used for diagnosis. Most research for 3D medical images have used 2D slices applied to CNNs. They require selecting meaningful 2D slices as part of pre- or post-processing to perform segmentation or detection of the liver^12^ or brain tumors^13^ from 3D data. This is time-consuming and difficult because it demands manual processing to get credible results: When 2D slice image data is used, the outputs of the 2D CNN will be as many as the number of slices, so the diagnosis result must be derived through some process that combines the different results into a single outcome. Moreover, although it is possible to extend CNNs into 3D to learn and extract features from 3D volumes, 2D slice-based CNN methods are susceptible to missing meaningful features found in 3D volume data.

**Figure 1:**
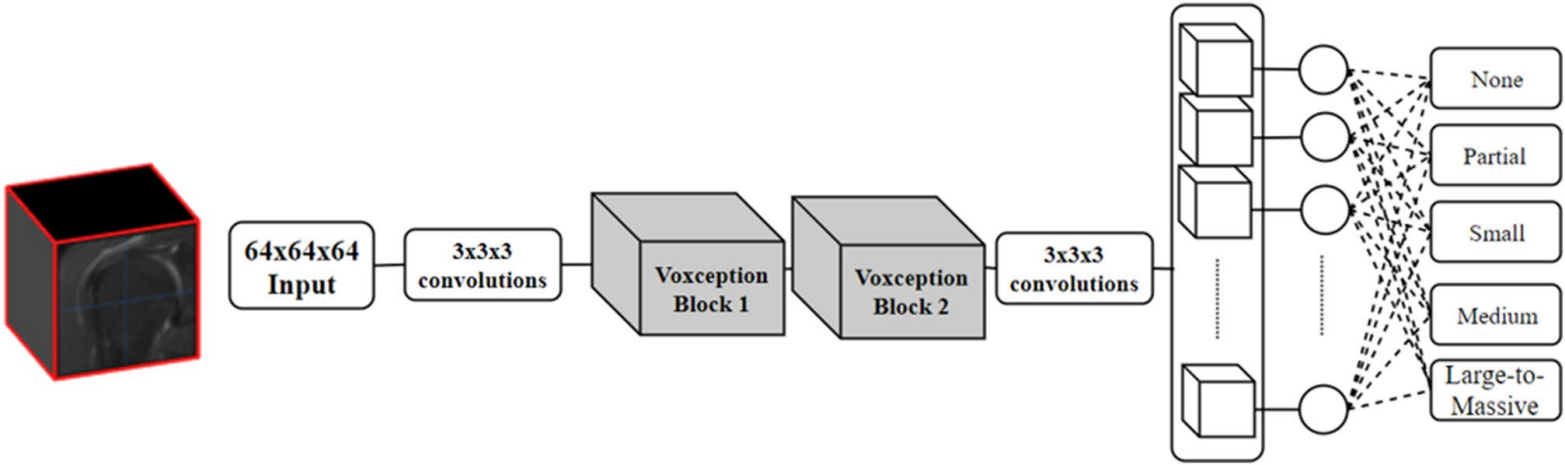
Network structure of the proposed method for automated rotator cuff tear (RCT) diagnosis. Original shoulder MRI volume is cropped and sampled into 64 × 64 × 64 volume. The sampled volume is feed-forwarded to the VRN-based 3D CNN to classify the RCT case (None, Partial, Small, Medium, and Large-to-Massive).

To overcome these problems, we propose a deep 3D-CNN method to classify rotator cuff tear (RCT), one of the most common shoulder injuries. RCT can be caused by the rupture of rotator cuff tendons, most frequently around the supraspinatus muscle, which causes pain and disability including weakness and decreased range of motion in the shoulder joint. The diagnosis of a RCT is largely based on patient’s symptoms, and accurate diagnosis is carried out using medical images; MRI data are currently widely used to diagnose RCTs. Once the MRI data of a patient’s shoulder is obtained, a skilled clinician identifies the site of rupture from several slice images and determines the presence, absence, and size of the rupture as well as the necessity of operation. While there is still controversy, automated deep-learning-based diagnosis is accepted for enhancing clinicians’ diagnostic performance in terms of accuracy and speed. Our proposed method utilizes the 3D information of a complete shoulder MRI volume, and performs the entire RCT diagnosis instantaneously. Unlike most existing previous CNN-based diagnosis methods, our method does not require intensive preprocessing of training data. Only the patient’s diagnosis information (normal or RCT size) and simple region of interest (ROI) selection are needed for preprocessing. Because the size of the RCT is measured during arthroscopic surgery, labeling results were credible in our retrospective study training data. Furthermore, our method uses fairly deep 3D CNN: three-dimensionally extended inception-ResNet. This network can extract meaningful 3D features from raw volume data for diagnosis by weakly-supervised learning. We also propose an effective visualization method using 3D volume rendering of class activation map (CAM)^14^. As a result, the proposed method automatically determines the RCT size in five categories (None, Partial, Small, Medium, Large-to-Massive) and visualizes the 3D localization information of RCT as a second opinion for clinical decision (Fig. 1).

## Results

For quantitative evaluation, a total of 2,124 patient MRI data were obtained. Among 2,124 MRI data sets, 200 were randomly selected as the test set, and the remaining 1,924 were used as the training set. The distribution of each category is listed in Table 1. We used and trained the VRN-based 3D CNN using these test and training sets to classify the subject data. All training were conducted until epoch 100. All the hyperparameter values were set based on Brock et al.^15^.

**Table 1:** MRI data sets used for training and test: all data were categorized according to the tear size.

Class	Train	Test	Total	
None	692	72	764	
**RCT**				
Partial	254	31	285	1360
Small	205	22	227	
Medium	518	49	567	
Large	225	26	281	
Total	1924	200	2124	

### Evaluation of the 3D CNN algorithm

The performance of the 3D CNN for diagnosing RCTs was evaluated for binary accuracy, sensitivity, specificity, precision and F1-score, and that for classifying RCTs was evaluated for top-1 accuracy, top-1 ± 1 accuracy, sensitivity, specificity, and diagnostic time. The values of top-1 ± 1 accuracy were defined by regarding one-size prediction error as correct. For example, when the predicted tear category was “Medium”, and the ground truth was “Small” or “Large”, it was counted as the correct prediction in the top-1 ± 1 accuracy metric.

### Evaluation of the diagnostic performance of human readers

**Figure 2:**
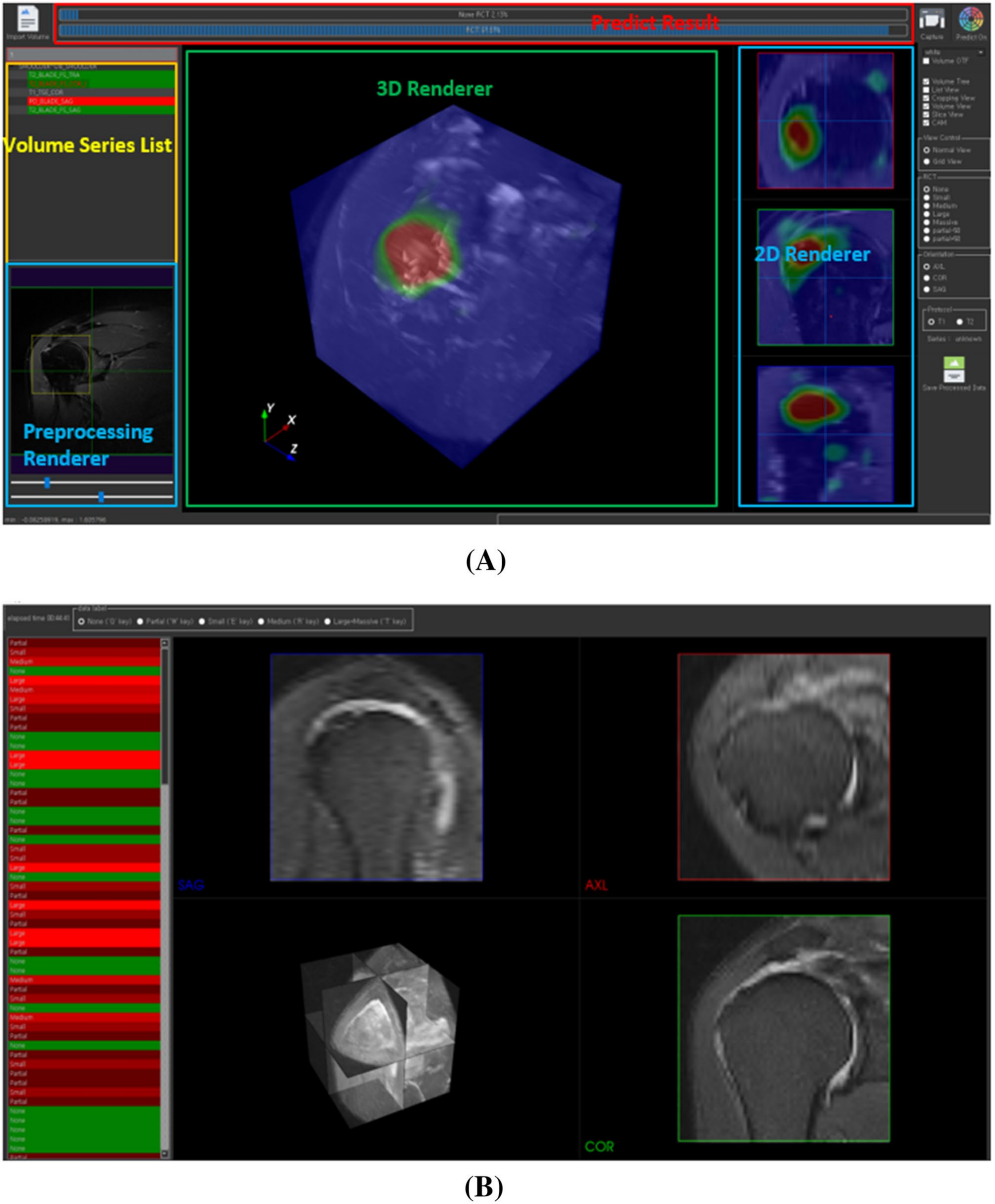
(**A**) A screen shot of the developed software for automated diagnosis and 3D visualization of RCT by the VRN-based 3D CNN. (**B**) In-house labeling software for comparative study with clinical expert. Seventeen orthopedic doctors classified the RCT of 200 test data sets using this software.

We developed a labeling software for comparing the performance of diagnosis and classification of RCT of 3D CNN with those of human readers (Fig. 2). The human readers comprised 13 general orthopedists and 4 orthopedic shoulder specialists. Each reader was requested to provide the most probable diagnosis of each MRI of the same 200 test data set of the 3D CNN. The human readers can choose one among normal,partial tear, small tear, medium tear, or large-to-massive tear, after reviewing the entire MRI data by using the developed labeling software. Subsequently, we calculated the top-1 accuracy, top-1±1 accuracy, sensitivity, specificity, and diagnostic time along with those of the 3D CNN, and we then compared the values.

### Statistics

All statistical analysis were performed using SPSS 15.0 (SPSS, Inc., Chicago, IL, USA). Descriptive statistics were used to report each value of the binary accuracy, top-1 accuracy, top-1 ± 1 accuracy, sensitivity, specificity, precision, F1-score, and diagnostic time.

**Figure 3:**
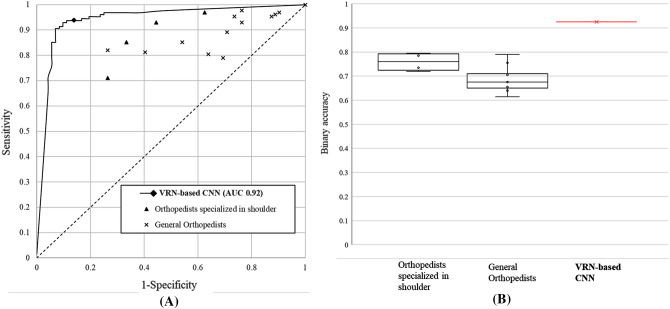
Comparison of diagnosis performance between clinical experts and the proposed method. (**A**) Receiver operating characteristic (ROC) curve of the proposed method, and the sensitivity-specificity distribution of each clinical expert; (**B**) Binary accuracy of each group.

For binary classification of RCT (Table 2 and Fig. 3), the VRN-based CNN exhibited excellent diagnostic performance with an accuracy 92.5%, sensitivity of 0.94, specificity of 0.90, precision of 0.94, and F1-score of 0.94. The results for the orthopedic shoulder specialists indicated an average binary accuracy of 76.4%, sensitivity of 0.86, specificity of 0.58, precision of 0.79, and F1-score of 0.82, and those of the general orthopedists showed an average binary accuracy of 68.2%, sensitivity of 0.90, specificity of 0.29, precision of 0.70, and F1-score of 0.78.

**Table 2:** RCT diagnosis performance of 3D CNN for binary classification.

	VRN-based 3D CNN
Binary accuracy	92.5% ± 1.36%
Sensitivity	$0.94 \pm 0.03$
Specificity	$0.90 \pm 0.04$
Precision	$0.94 \pm 0.02$
F1-score	$0.94 \pm 0.01$

**Figure 4:**
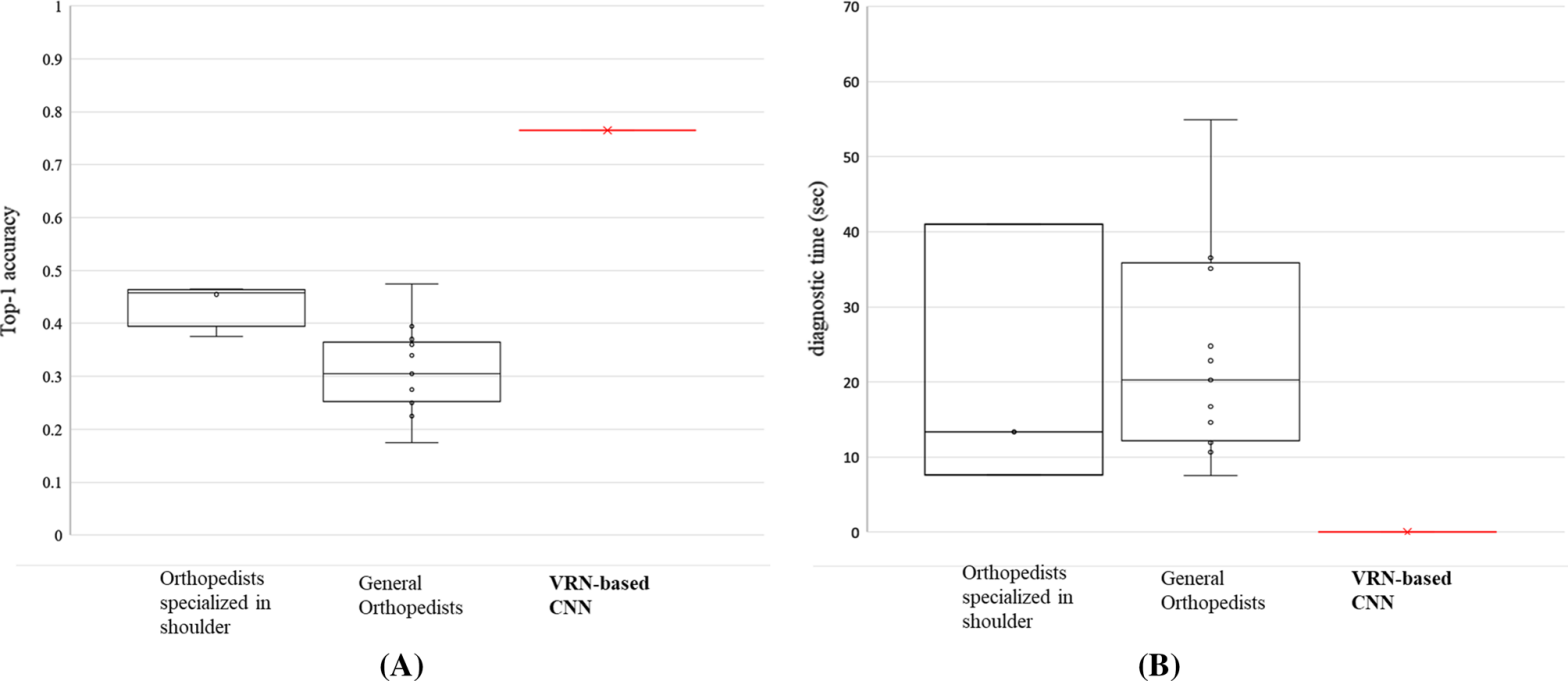
Comparison of diagnostic performance between clinical experts and the proposed method. (**A**) Top-1 accuracy; (**B**) diagnostic time.

For the multi-class RCT classification, the performance results for each group were summarized as a median with first & third quartiles) (Table 3). Also, the performance of the 3D CNN was compared with those of each human group using the statistical analysis of Kruskal-Wallis test, followed by Bonferroni post hoc analysis with the significance level set at p < 0.0167. The VRN-based 3D CNN repeated the test 60 times for the same 200 test-set data, and the mean value of the 60 repetitions were used. As shown in Table 3a, the VRN-based CNN achieved top-1 accuracy of 69.0% and top 1 ± 1 accuracy of 87.5%; whereas the orthopedic shoulder specialists achieved top-1 accuracy of 45.8% and top 1±1 accuracy of 79.8%; and the general orthopedists had top-1 accuracy of 30.5% and top 1±1 accuracy of 71.0% as a median value. The VRN-based 3D CNN took only 0.01 s diagnostic time to classify one case. However, the orthopedic shoulder specialists and general orthopedists took 20.3 s and 34.2 s, respectively, to achieve the same tasks (Fig. 4b). According to the results of Bonferroni pot-hoc analysis for the performance for RCT classification between groups (Table 3b–f), statistically significant p-values were marked for Top-1 accuracy, Top-1 ± 1 accuracy, specificity, and diagnostic time.

**Table 3:** Comparison of the performance for multi-class RCT classification between groups.

(a)	VRN-based 3D CNN	Orthopedic shoulder specialist	General orthopedist	p-value
Top-1 accuracy	69.0 (67.0, 70.1)%	45.8 (41.0, 46.4)%*	30.5 (25.3, 36.5)%*	$< 0.001$
Top-1 ± 1 accuracy	87.5 (86.5, 88.5)%	79.8 (75.9, 86.3)%*	71.0 (66.0, 76.3)%*	$< 0.001$
Sensitivity	0.92 (0.90, 0.94)	0.89 (0.75, 0.96)	0.93 (0.82, 0.96)	0.778
Specificity	0.86 (0.83, 0.89)	0.61 (0.42, 0.72)*	0.26 (0.12, 0.41)*	$< 0.001$
Diagnostic time (s)	0.01 (0.01, 0.01)	20.3 (12.2, 35.9)*	34.2 (9.1, 219.4)*	$<0.001$

	VRN-based 3D CNN	Orthopedic shoulder specialist	General orthopedist	
**(b) Top-1 accuracy**				
VRN-based 3D CNN		$< 0.001*$	$<0.001*$	
Orthopedic shoulder specialist	$<0.001*$		$0.010*$	
General orthopedist	$< 0.001*$	$0.010*$		
**(c) Top-1 ± 1 accuracy**				
VRN-based 3D CNN		0.010*	$<0.001*$	
Orthopedic shoulder specialist	$0.010*$		0.023	
General orthopedist	$<0.001*$	0.023		
**(d) Sensitivity**				
VRN-based 3D CNN		0.491	0.938	
Orthopedic shoulder specialist	0.491		0.624	
General orthopedist	0.938	0.624		
**(e) Specificity**				
VRN-based 3D CNN		$<0.001*$	$<0.001*$	
Orthopedic shoulder specialist	$<0.001*$		0.023	
General orthopedist	$<0.001*$	0.023		
**(f) Diagnostic time (s)**				
VRN-based 3D CNN		$< 0.001*$	$<0.001*$	
Orthopedic shoulder specialist	$< 0.001*$		0.703	
General orthopedist	$< 0.001*$	0.703		

(a) The performance values were described as a median (first quartile, third quartile) and the p-values indicate the results of Kruskal-Wallis test. (b–f) The results of Bonferroni post hoc analysis (*Statistically significant, p < 0.0167).

## Discussion

We demonstrated the robustness of the proposed method in diagnosis of RCT MRI data by the evaluation test comparing with clinical experts. The experiment showed that the trained VRN-based 3D CNN outperforms human orthopedists in binary accuracy, sensitivity, specificity, precision, f1-score, top-1 accuracy, top-1±1 accuracy, and diagnostic time. In comparative experiment results including clinical experts, the proposed method performed the best for all metrics. In binary accuracy results, it achieved 16.1% and 24.3% higher than orthopedists specialized in shoulder and general orthopedists. The results of sensitivity and specificity also proved the proposed method’s robustness. In the results of clinical experts, specificity tends to be low (0.58 for orthopedists specialized in shoulder; 0.29 for general orthopedists). The proposed method showed higher sensitivity (0.94) and specificity (0.90) than clinical experts. All the human expert results were under the VRN-based 3D CNN’s receiver operating characteristic (ROC) curve (Fig. 3). In top-1 accuracy, the proposed method resulted in 23.2%, 38.5% higher than clinical experts. In top-1 ± 1 accuracy, the proposed method showed 7.7% and 16.5% higher than orthopedists specialized in shoulder and general orthopedists. The results were more dramatic when comparing the diagnostic time. Even we provided the participating clinical experts with specially-designed convenient in-house software for the evaluation test, it took more than 20 seconds per one case. In contrary, it took less than two seconds to diagnose 200 test data (0.01 s per one case in average) by the proposed method. These results indicates that the proposed method can reduce diagnostic time while supporting the accurate diagnosis of RCT.

**Figure 5:**
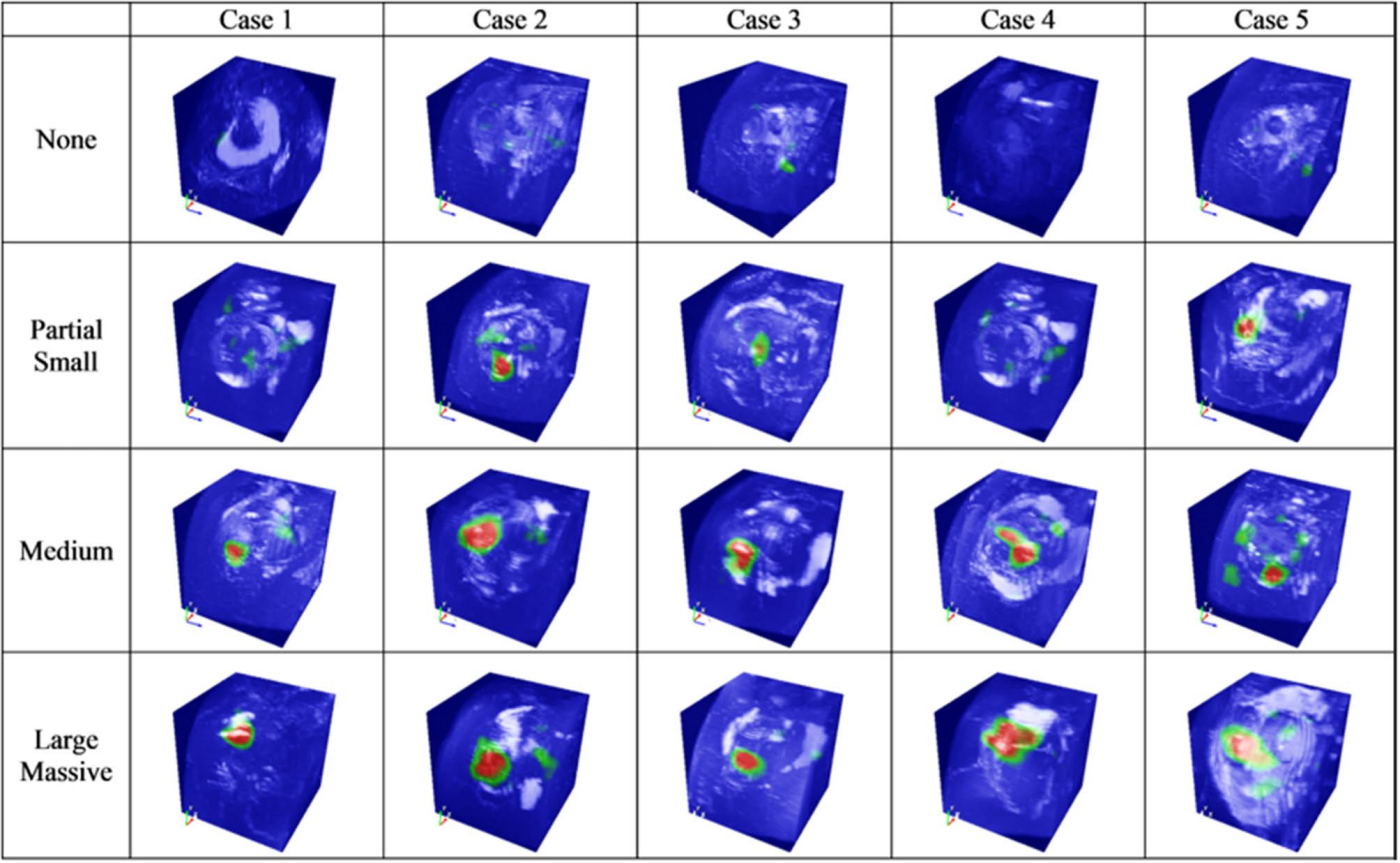
Sample cases for of each tear size with colored heatmap visualization by volume rendering of 3D class activation map (CAM), which shows 3D localization and size of RCT.

**Figure 6:**
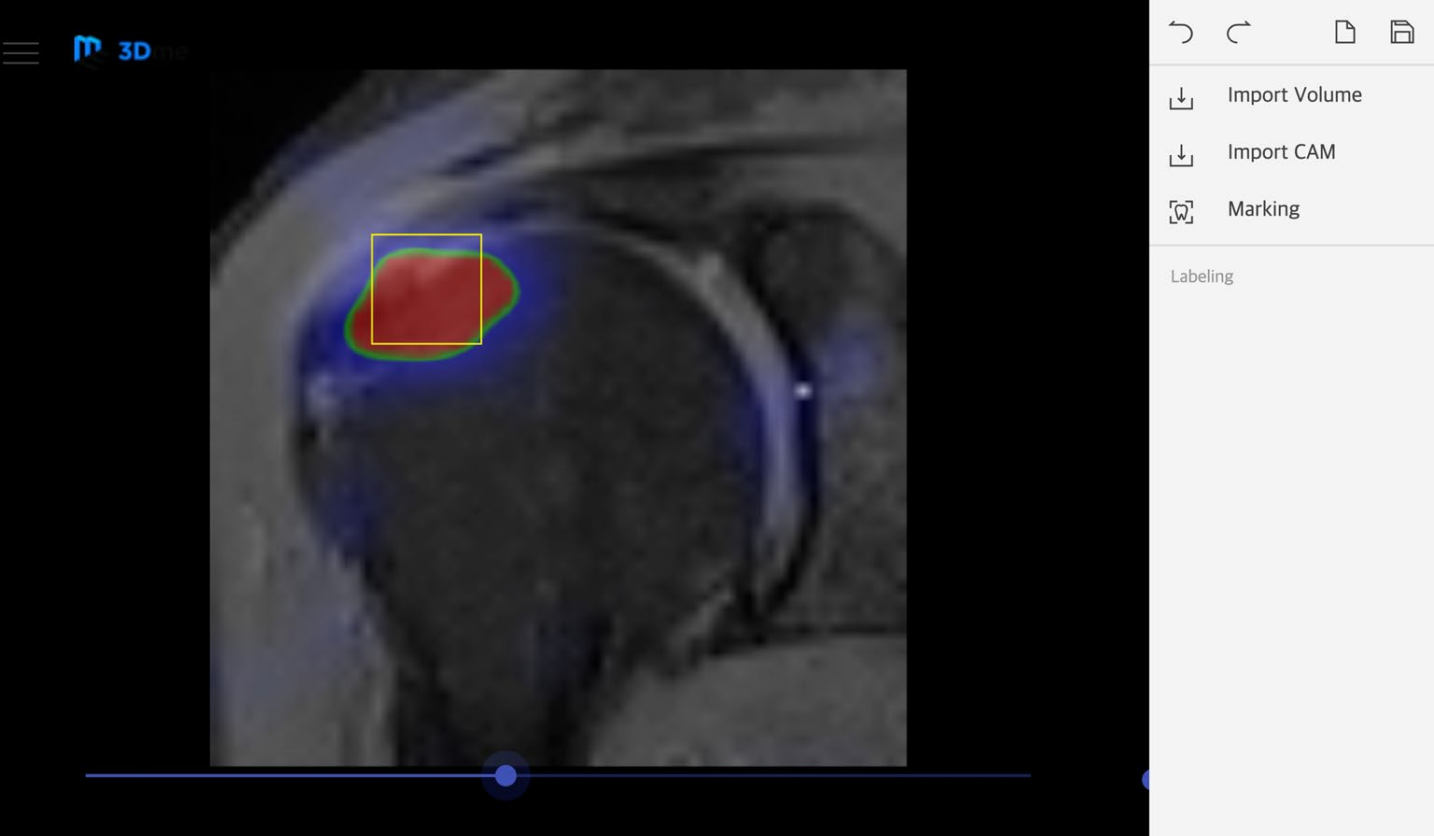
An example which shows the visualized CAM location is on the same position of RCT where an orthopedic shoulder specialist marked on the original MR image with a yellow box.

Furthermore, using class activation map (CAM) method^14^, we could visualize the location and size of RCT from 3D MRI data. Because our deep learning architecture is 3D convolution filter-based 3D CNN, the generated CAM is also 3D volume data. By using volume rendering method, colored heatmap can be visualized, and the user can visually check the approximate location and size of RCT (Fig. 5). To evaluate the CAM visualization results, two orthopedic shoulder specialists checked the visualized RCT locations by CAM for all MRI data in this study.By using in-house software, we validated whether the CAM location coincides with the blinded orthopedic shoulder specialist’s RCT marking (Fig. 6). It was confirmed that all the CAM locations were the same as the actual RCT locations, and there was no incorrect case.

In addition to this, 3D convolution filters can use spatial information from neighboring slices. The results of the experiment indicate that the VRN-based 3D CNN effectively uses 3D features from the MRI volume input for classification of RCT. The colored heatmap 3D CAM visualization, which highlights only the RCT area, demonstrates the effectiveness of proposed method.

Another advantage of the proposed method is that preprocessing of the training data is simple. The ground truth data were labeled with only minimal information: normal or size of the RCT. The tear size was easily obtained from retrospective study because it had been measured and recorded in the patient’s chart during arthroscopic surgery. For training, only the 3D MRI data and this categorical size of RCT and 3D were provided to the VRN-based 3D CNN. There was no information of RCT location of shape used for training. After training, the proposed method yields not only classification results, but also the 3D location and shape information in 2D slice and 3D. This can be considered weakly-supervised learning, which provides more representative feature information (location and shape of RCT) that was not included in the ground-truth training data. This weakly-supervised deep learning method is easy to apply while giving accurate clinical results and 3D visualized localization information about the injury.

To our knowledge, this is the first study of a deep learning-based method applied to tendon tear diagnosis. Also, this is the first study that uses deep 3D CNN for diagnosis of a large MRI volume. This method enables 3D CAM visualization, providing the 3D location and size for the area of interest. Using automated diagnostic results and 3D visual information as a second opinion, it is expected that diagnostic accuracy and time savings for clinical experts will be improved. In the future, it is expected that the performance can be improved further; as CNN training is data-driven, having a larger amount of training data will improve its performance and reliability. Recent CNN structures such as densely connected convolutional networks^16^ or squeeze-and-excitation networks^17^ could also be used to try to improve performance.

Additionally, comparative experiments with existing 2D-based CNN methods need to be performed in the future. Theoretically, we can expect the robustness of 3D CNN in terms of data preprocessing and labeling, feature extraction during training, and feed-forwarding time in prediction, but direct comparative validation with existing methods is needed. No matter how well the performance is verified, computer-assisted automatic diagnosis should always provide some kind of justification of its decision. Because the trained neural network is considered a “black box”, the 3D CAM visualization is important for real-world application. We proposed an end-to-end 3D CNN method to classify 3D MRI data, and this novel approach enables not only successful training on 3D features for classification but also generation of 3D CAM. The visualized 3D data can offer rich information compared with traditional 2D-based methods and the 3D CAM generated by a well-trained VRN can indicate the location of an RCT. Currently, evaluation of CAM’s localization or shape generation was not numerically measured. Obtaining the ground truth of localization or shape, and evaluation of CAM will be conducted as future works.

## Methods

### Data preparation

 Retrospective shoulder MRI data from 2,124 patients were gathered at Konkuk University Hospital, South Korea. The study protocol was approved by the ethics committee of Konkuk University Hospital (IRB No. KUH1060168) with a waiver of informed consent. The data were labeled according to the diagnosis status of the rotator cuff and its tear size: (None, Partial, Small, Medium, and Large-to-Massive). The tear size was determined based on the tear size measured using a calibrated probe during arthroscopic surgery by a senior author (S.W.C.) and classified according to the rating systems of DeOrio and Cofield. (small size < 1 cm, medium size: 1 to 3 cm, large-to-massive size > 3 cm)^18^. All data in the “None” category were also visually ascertained via arthroscopic surgery. The “None” MRI data were obtained from patients not because of rotator cuff tears but for other reasons such as SLAP lesion or shoulder instability. No exact locations were labeled. Each MRI scan was obtained on a 3.0-T system (Signa HDx; GE Healthcare) with a dedicated shoulder coil. The following MRI sequences were used: axial images obtained with T1-weighted spin echo (repetition time/echo time: 550-733/15-17 ms) sequences, and coronal and sagittal images obtained with T2-weighted spin echo (repetition time/echo time: 3500-4000/60-110 ms) sequences. The slice thickness was 4 mm, with a slicing gap of 0 or 0.4 mm and a field of view of $16 \times 16$ cm. All preprocessing data were humeral-head-centered, and cropped into cube, normalized from 0 to 255 greyscale.

### Training

 Voxception-ResNet (VRN) is three-dimensionally extended network architecture of Inception-ResNet. The original model was developed for classification of voxelated 3D mesh objects^15^. A voxel in 3D volume is a pixel in 2D image defined in three dimensions, and it is the basic unit in 3D volume. Each Voxception block is composed of three Voxception-ResNet structure and one Voxception-Downsample structure. Voxception-ResNet structure uses Voxception structure that contains (3 × 3 × 3) and (1 × 1 × 1) 3D convolution filter and residual connection. The Voxception-Downsample structure reduces the dimension using a Voxception structure with 3D convolution with 2 strides, and pooling. We have modified this structure to perform MRI volume classification and CAM visualization. First, the input size was extended to (64 × 64 ×  64) from (32 × 32 × 32) to prevent excessive information loss. Second, the second fully connected layer was removed in order to generate the CAM. According to Zhou et al.,^14^, this modification of network architecture does not have a significant impact on performance. Third, the number of Voxception-blocks were reduced from four to two. Through this structure change, it was possible to obtain a higher resolution CAM. For the experiment, the VRN-based CNN has five classes as output for multi-class classification (None, Partial, Small, Medium, Large-to-Massive). For training, cross-entropy loss was computed from the softmax output and the ground truth. Back propagation with stochastic gradient descent by using the loss value was employed to optimize the trainable variables of the network. Nesterov momentum was applied with a momentum value of 0.9, and Xavier’s initialization method was applied to initialize the network’s trainable weight parameters. The learning rate was initially set to 0.002 and reduced to 0.0002 after epoch 12. Batch size was set to 6; batch normalization^19^, L2 regularization, and dropout were applied to improve the test performance and avoid problems such as vanishing gradients and overfitting. Additionally, the training data order was shuffled in every epoch. Training was conducted to epoch 100. The hyperparameter was fixed as mentioned above, and no validation set was used for fine-tuning the hyperparameter.

### Integrated software

 We implemented an integrated software program (Fig. 2), which supports Dicom data import, visualization, and preprocessing. Moreover, the trained network is built in, and RCT classification and 3D visualization of CAM are also supported. This in-house software was developed in Python 3.x and the QT framework was used to build the graphical user interface. Insight ToolKit (ITK) and Visualize ToolKit (VTK) libraries are used to process and visualize the volume data.
