# Author Correction: Automated rotator cuff tear classification using 3D convolutional neural network

**DOI:** 10.1038/s41598-021-95469-7

**Published:** 2021-08-02

**Authors:** Eungjune Shim, Joon Yub Kim, Jong Pil Yoon, Se‑Young Ki, Taewoo Lho, Youngjun Kim, Seok Won Chung

**Affiliations:** 1 Center for Bionics, Korea Institute of Science and Technology, Seoul, 02792 Korea; 2 Department of Orthopedic Surgery, Yeson Hospital, Bucheon, 14555 Korea; 3 Department of Orthopaedic Surgery, School of Medicine, Kyungpook National University, Daegu, 41944 Korea; 4 Department of Orthopaedic Surgery, Center for Shoulder and Elbow Surgery, Konkuk University School of Medicine, Seoul, 143‑729 Korea

Correction to: *Scientific Reports* https://doi.org/10.1038/s41598-020-72357-0, published online 24 September 2020

The original version of this Article contained errors in the Abstract.

“The VRN-based 3D CNN outperformed orthopedists specialized in shoulder and general orthopedists in binary accuracy (92.5% vs. 76.4% and 68.2%), top-1 accuracy (69.0% vs. 45.8% and 30.5%), top-1±1 accuracy (87.5% vs. 79.8% and 71.0%), sensitivity (0.94 vs. 0.86 and 0.90), and specificity (0.90 vs. 0.58 and 0.29).”

now reads:

“The VRN-based 3D CNN outperformed orthopedists specialized in shoulder and general orthopedists in binary accuracy (92.5% vs. 76.4% and 68.2%), top-1 accuracy (69.0% vs. 45.8% and 30.5%), top-1±1 accuracy (87.5% vs. 79.8% and 71.0%), sensitivity (0.92 vs. 0.89 and 0.93), and specificity (0.86 vs. 0.61 and 0.26).”

The original Article has been corrected.

